# A GM-CSF/IL-33 Pathway Facilitates Allergic Airway Responses to Sub-Threshold House Dust Mite Exposure

**DOI:** 10.1371/journal.pone.0088714

**Published:** 2014-02-14

**Authors:** Alba Llop-Guevara, Derek K. Chu, Tina D. Walker, Susanna Goncharova, Ramzi Fattouh, Jonathan S. Silver, Cheryl Lynn Moore, Juliana L. Xie, Paul M. O’Byrne, Anthony J. Coyle, Roland Kolbeck, Alison A. Humbles, Martin R. Stämpfli, Manel Jordana

**Affiliations:** 1 Department of Pathology and Molecular Medicine, McMaster Immunology Research Centre, Hamilton, Ontario, Canada; 2 Department of Respiratory, Inflammation and Autoimmunity, MedImmune LLC, Gaithersburg, Maryland, United States of America; 3 Department of Medicine, McMaster University, Hamilton, Ontario, Canada; 4 Pfizer, Cambridge, Massachusetts, United States of America; National Jewish Health, United States of America

## Abstract

Allergic asthma is a chronic immune-inflammatory disease of the airways. Despite aeroallergen exposure being universal, allergic asthma affects only a fraction of individuals. This is likely related, at least in part, to the extent of allergen exposure. Regarding house dust mite (HDM), we previously identified the threshold required to elicit allergic responses in BALB/c mice. Here, we investigated the impact of an initial immune perturbation on the response to sub-threshold HDM exposure. We show that transient GM-CSF expression in the lung facilitated robust eosinophilic inflammation, long-lasting antigen-specific Th2 responses, mucus production and airway hyperresponsiveness. This was associated with increased IL-33 levels and activated CD11b^+^ DCs expressing OX40L. GM-CSF-driven allergic responses were significantly blunted in IL-33-deficient mice. IL-33 was localized on alveolar type II cells and *in vitro* stimulation of human epithelial cells with GM-CSF enhanced intracellular IL-33 independently of IL-1α. Likewise, GM-CSF administration *in vivo* resulted in increased levels of IL-33 but not IL-1α. These findings suggest that exposures to environmental agents associated with GM-CSF production, including airway infections and pollutants, may decrease the threshold of allergen responsiveness and, hence, increase the susceptibility to develop allergic asthma through a GM-CSF/IL-33/OX40L pathway.

## Introduction

Allergic asthma is a complex immune-driven disease of the airways that may develop in susceptible individuals in response to aeroallergen exposure. While allergen exposure is universal, the prevalence of allergic sensitization and asthma is 35% and 10%, respectively [1], [2]. The dramatic increase in the prevalence of asthma over the last five decades in Western countries [2] intimates the contribution of factors other than genetic predisposition to the development of asthma. Given that allergen exposure often occurs concurrently with exposures to biologicals (*e.g.* viruses, bacteria) and/or chemicals (*e.g.* pollution), environmental exposures affecting the immune status of the lung could lower the threshold of allergen responsiveness and precipitate the onset of the allergic diathesis.

We have previously provided a comprehensive computational view of the impact of dose and length of exposure on allergic responses [3]. This analysis precisely identified system parameters such as distinct thresholds for different inflammatory and immunological variables. Here, we set out to investigate *whether* and *how* a change in the immune status of the lung at the time of incipient allergen exposure would impact subsequent immunological and physiological responses. As a defining characteristic of complex systems is their sensitivity to initial conditions [4], we introduced a perturbation to transiently influence key initiating immunological events, namely the state of the antigen-presenting cell (APC) compartment. Using an adenoviral vector approach, we overexpressed GM-CSF in the airway only during early exposure to HDM. GM-CSF is a powerful natural cytokine able to stimulate the proliferation, maturation and function of APCs [5].

Our data show that transient expression of physiologically relevant levels of GM-CSF in the lung lowers the threshold of allergen required to generate allergic airway inflammation and dysfunction by at least ten times. This is associated with a marked increase in the number of activated CD11b^+^ dendritic cells (DCs) in the lung. Mechanistically, we show that GM-CSF significantly increases IL-33 expression by alveolar type II cells (ATII), and that GM-CSF-driven responses, including lung eosinophilia, Th2 cytokine production and expression of the critical co-stimulatory molecule OX40 ligand (OX40L), are substantially blunted in IL-33-deficient mice. These findings suggest that a wide array of environmental exposures at the time of initial contact with aeroallergens may increase the susceptibility to develop allergic asthma through a GM-CSF/IL-33/OX40L pathway.

## Results

### GM-CSF Facilitates and Exacerbates Inflammatory Responses to HDM

**Figure 1:**
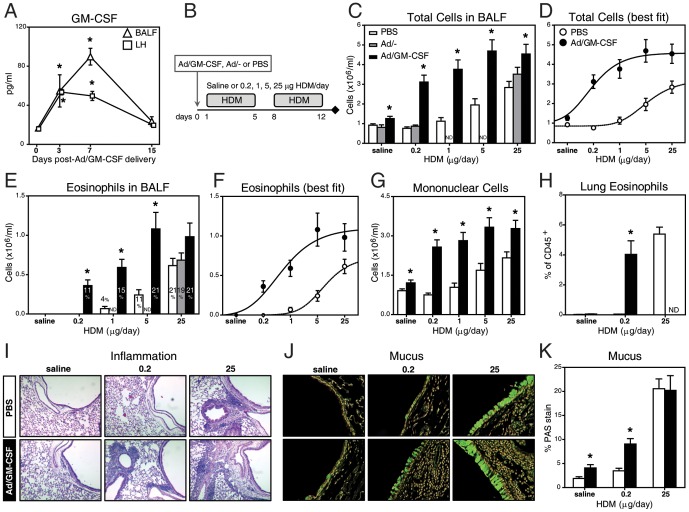
GM-CSF in the lung facilitates and exacerbates HDM responses. (**A**) GM-CSF by ELISA in BALF and lung homogenates (LH) from naïve mice (time 0) or after Ad/GM-CSF delivery. (**B**) Schematic of the protocol followed in **C-K** (see MATERIALS AND METHODS). (**C**) Inflammatory cell counts in BALF. (**D**) Best-fit curves for the original data in C. (**E**) Eosinophil numbers (bars) and percentages in BALF, as well as (**F**) best-fit curves. (**G**) Number of mononuclear cells in BALF. (**H**) Proportion of eosinophils (CD45^+^SiglecF^+^CD11b^+^CD11c^-^FSC^low^SSC^hi^) in the lung tissue by flow cytometry. Representative lung sections stained with (**I**) H&E (x100 magnification) or (**J**) PAS (x400, color-inverted). (**K**) Morphometric quantification of PAS staining. N = 11–18 mice/group. * p<0.05 in Ad/GM-CSF versus respective PBS group. ND for not determined.

We have previously reported that daily exposure to 0.2, 1, 5 and 25 µg of HDM induces absent, incipient, moderate and severe immune-inflammatory responses, respectively, in mice [3]. Here, we sought to examine the effect of GM-CSF on the responsiveness to HDM. In order to overexpress GM-CSF in the lung, mice were instilled intranasally with 3×10^7^ plaque-forming units (pfu) of a replication-deficient adenovirus encoding a mouse GM-CSF transgene (Ad/GM-CSF). Consistent with our previous reports [6], [7], GM-CSF expression peaked in the bronchoalveolar lavage fluid (BALF, ∼90 pg/ml) and lung homogenates (∼50 pg/ml) at 3 to 7 days after Ad/GM-CSF delivery and sharply decreased by 2 weeks, remaining very low and comparable to the levels detected in the empty Ad (Ad/−) and PBS treated groups (**Fig. 1A** and data not shown). GM-CSF was not detected in peripheral blood (not shown). Hence, this adenoviral vector approach leads to transient and compartmentalized GM-CSF release in the lung.

Allergic airway inflammation was evaluated after 2 weeks of intranasal HDM exposure, a time-point at which inflammation reaches the maximal level [3]. Here, Ad/GM-CSF, Ad/− or PBS were given 24 h before daily exposure to saline or increasing doses of HDM (schematic in **Fig. 1B**). As shown in **Fig. 1C**, HDM alone elicited airway inflammation in a dose-dependent manner with increased number of inflammatory cells starting at 1–5 µg HDM and reaching a plateau at 25 µg, in agreement with our previous studies [3]. In the presence of GM-CSF, inflammation was significantly increased across all HDM doses, including an almost 4-fold increase in mice exposed to 0.2 µg HDM. It is important to note that 0.2 µg HDM is a sub-threshold dose that does not induce any detectable immune-inflammatory response *per se*. In addition, five times less HDM (*i.e.* 5 µg) was required to elicit maximal responses in mice expressing GM-CSF. The best fit curves in **Fig. 1D** clearly show increased sensitivity (left shift), reactivity (greater slope) and maximal inflammatory responses in Ad/GM-CSF versus PBS-treated mice. These increases were mainly due to airway eosinophils (**Fig. 1E–F**) and mononuclear cells (**Fig. 1G**). Similarly, lung eosinophils (CD45^+^SiglecF^+^CD11b^+^CD11c^-^) were significantly elevated in mice exposed to Ad/GM-CSF along with 0.2 µg HDM (heretofore referred as Ad/GM-CSF+0.2) (**Fig. 1H**). In all groups receiving Ad/GM-CSF, neutrophils were slightly increased (<5%) as compared to the PBS controls (∼1%, not shown). Tissue inflammation (**Fig. 1I**) paralleled BALF data. Peribronchial and perivascular inflammation was clearly observed in mice exposed to Ad/GM-CSF+0.2 but absent in the HDM alone group. We also examined goblet cell hyperplasia and hypertrophy in PAS-stained lung sections (**Fig. 1J**). A morphometric analysis of the main airway epithelium revealed significantly greater mucus production in mice exposed to Ad/GM-CSF+0.2 compared to PBS+0.2 (**Fig. 1K**). Thus, these findings show that GM-CSF overexpression facilitates and synergistically exacerbates inflammatory responses to HDM. From this point forward, we focused our studies on the effects of the sub-threshold dose of HDM.

### GM-CSF Promotes Type 2 Immunity to Sub-threshold HDM Exposure

**Figure 2:**
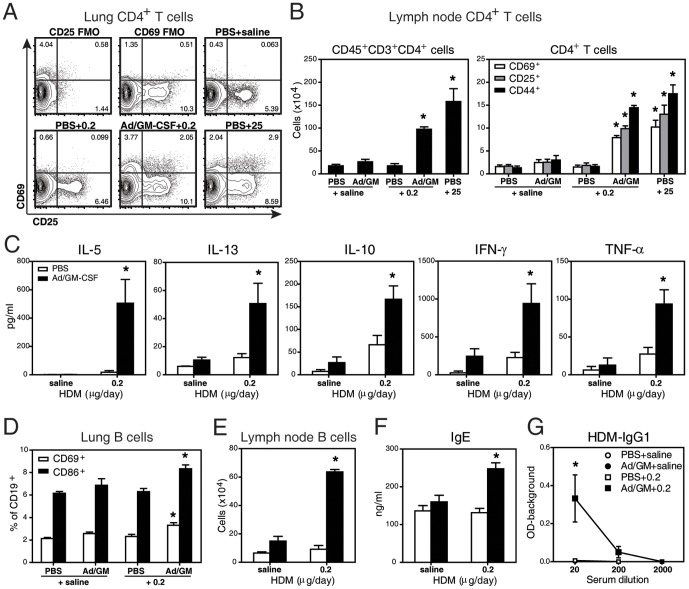
GM-CSF promotes type 2 immunity to HDM. (**A**) Representative flow plots of CD4^+^ T cells (CD45^+^CD3^+^CD19^-^) showing expression of CD69 and CD25 in the lung. (**B**) Numbers of total, CD69^+^, CD25^+^ and CD44^+^ CD4^+^ T cells in thoracic lymph nodes. (**C**) Cytokine levels in supernatants of splenocytes stimulated *in vitro* with HDM. (**D**) Proportion of B cells (CD45^+^CD3^-^CD19^+^) that are CD69^+^ or CD86^+^ in the lung. (**E**) B cell numbers in the lymph node. Levels of (**F**) total IgE and (**G**) HDM-specific IgG1 in serum. All samples were collected at the end of the 2 week protocol (**Figure 1B**). N = 3–4 mice/group in **A,B,D,E** and N = 5–15 mice/group in **C,F**. * p<0.05 in Ad/GM-CSF+0.2 versus PBS+0.2 or PBS+25 versus PBS+saline values.

To examine whether these inflammatory changes involved an adaptive immune response, we evaluated lymphocyte populations in the lung and draining thoracic lymph nodes. **Fig. 2A–B** depicts increases in the proportion and number of activated, effector and memory CD4^+^ T cells (CD45^+^CD11c^-^CD3^+^CD4^+^ and CD69^+^, CD25^+^ or CD44^+^, respectively) in Ad/GM-CSF+0.2 exposed mice. Upon stimulation of splenocytes with HDM *in vitro*, Th2 cytokines (IL-5 and IL-13), IL-10, Th1 (IFN-γ) and TNF-α were significantly elevated in Ad/GM-CSF+0.2-treated mice (**Fig. 2C**), confirming an antigen-specific response.

Similarly, the number and proportion of activated B cells (CD45^+^CD3^-^CD19^+^ and CD69^+^ or CD86^+^) were greater in the presence of Ad/GM-CSF (**Fig. 2D–E**). B cell functionality, as assessed by the levels of serum IgE and HDM-specific IgG1, was also significantly elevated (**Fig. 2F–G**). Hence, a GM-CSF-enriched environment promotes allergic sensitization in mice receiving a sub-clinical dose of HDM (0.2 µg).

### Transient GM-CSF Overexpression Leads to HDM-specific Long-term Responses

**Figure 3:**
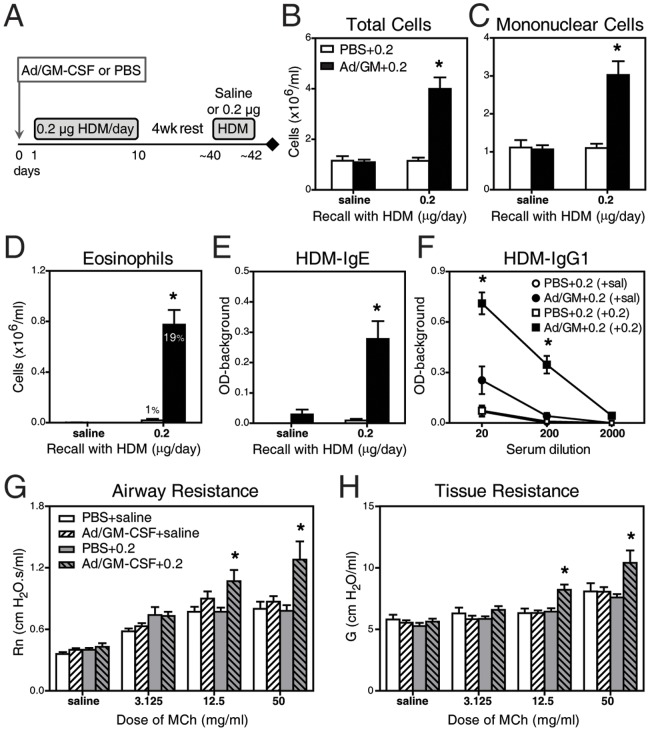
Initial GM-CSF leads to allergic long-term responses. (**A**) Protocol followed in B–F: mice received Ad/GM-CSF or PBS 24 h prior to HDM exposure for 10 d; then, mice were rested for 4 weeks (no allergen) before re-exposure to either saline or HDM for 3 days, with sample collection occurring 24 h after the last exposure. (**B–D**) Inflammatory cell numbers in BALF. Levels of (**E**) HDM-specific IgE and (**f**) HDM-specific IgG1 in serum. In (**G,H**), mice received Ad/GM-CSF or PBS and either saline or 0.2 µg HDM for 4 consecutive weeks, with 2 days rest in between each week. (**G,H**) Airway hyperresponsiveness based on peak values for Rn and G. N = 4–14 mice/group. * p<0.05 in Ad/GM-CSF+0.2 versus PBS+0.2.

We next sought to investigate the long-term impact of these early GM-CSF-induced immune-inflammatory changes. As shown in the diagram in **Fig. 3A**, mice received Ad/GM-CSF or PBS and 0.2 µg HDM for 10 consecutive days; then, allergen exposure was discontinued for 4 weeks until inflammation was resolved [8], at which time animals were re-exposed to saline or sub-threshold doses of HDM for 3 consecutive days. Note that such short exposure does not induce any detectable inflammatory response in naïve mice (not shown). As depicted in **Fig. 3B,D**, re-exposure with 0.2 µg HDM resulted in robust inflammation, rich in eosinophils, only in mice that initially received Ad/GM-CSF+0.2. The levels of HDM-specific IgE and IgG1 were also significantly elevated (**Fig. 3E–F**). Re-exposure of sensitized mice to a ten times lower dose of HDM (0.02 µg) already induced a mild inflammatory response with 4.3% eosinophils (not shown). As expected, animals initially receiving PBS+0.2 were not sensitized and, therefore, did not respond to subsequent HDM re-exposure. Thus, initial GM-CSF overexpression induces an antigen-specific immune response that maintains memory capacity resulting in robust immune-inflammatory responses after exposure to sub-threshold doses of allergen.

Another set of mice were daily exposed to HDM for 4 weeks to evaluate lung function. Only mice that received Ad/GM-CSF+0.2 exhibited greater airway hyperresponsiveness. In particular, airway (Rn, **Fig. 3G**) and tissue (G, **Fig. 3H**) resistance to nebulized methacholine (MCh) were significantly higher, reflecting greater bronchoconstriction and heterogeneity of small airway narrowing. These findings suggest that an early and transient perturbation of the immune system can have a long-lasting immunological and physiological impact after exposure to sub-clinical allergen doses.

### GM-CSF-induced Allergic Responses to HDM Require IL-33

In **Fig. S1**, we show that GM-CSF overexpression increased the number of total and activated CD11b^+^ DCs early on, arguably facilitating responsiveness to sub-threshold amounts of HDM.

**Figure 4:**
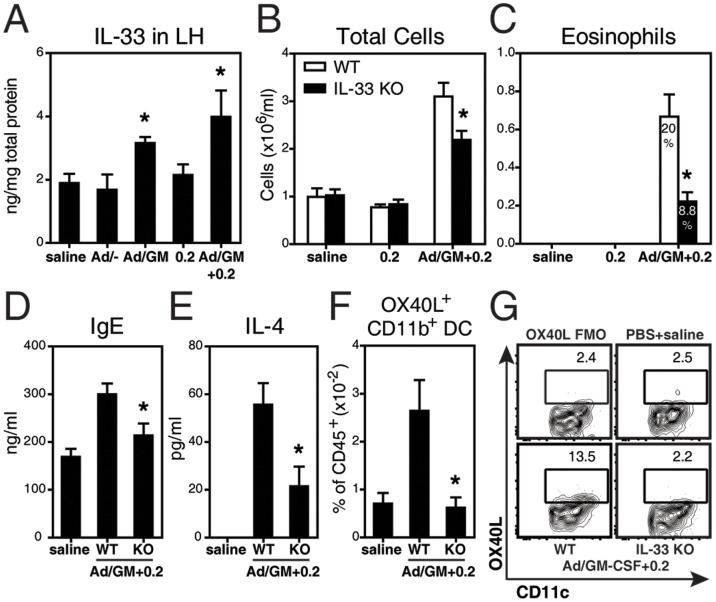
IL-33 mediates GM-CSF-driven responses to HDM. (**A**) IL-33 by ELISA in lung homogenates of WT mice at day 8. BALB/c WT and IL-33 KO mice were exposed to HDM for either (**B–E**) 2 weeks or (**F,G**) 7 consecutive days. (**B**) Total cells and (**C**) eosinophil numbers and percentages in BALF. (**D**) Total IgE in serum. (**E**) IL-4 production by splenocytes stimulated *in vitro* with HDM. (**F**) Proportion of OX40L^+^ CD11b^+^ DCs in the lung. (**G**) Representative flow plots showing expression of OX40L on DCs. N = 4–19 mice/group. * p<0.05 in (**A**) all groups versus saline, (**B–G**) IL-33 KO versus respective WT.

We have recently reported that exposure to 25 µg HDM, a dose that elicits maximal allergic responses, upregulates IL-33, and that IL-33 signalling is required for allergic sensitization in part via upregulation of the co-stimulatory molecule OX40L on DCs [9]. Here, we quantified IL-33 protein in lung homogenates and observed only baseline levels in mice receiving 0.2 µg HDM (**Fig. 4A**). Administration of Ad/GM-CSF and Ad/GM-CSF+0.2 increased the levels of IL-33 in the lung. IL-33 was not detected in the BALF (not shown). We next studied the relevance of IL-33 in this GM-CSF-driven model. As depicted in **Fig. 4B**, airway inflammation upon Ad/GM-CSF+0.2 delivery was significantly decreased in IL-33 KO mice compared to WT controls. In particular, IL-33 KO mice had over 60% less eosinophils (**Fig. 4C**) and IgE (**Fig. 4D**), indicating a marked dampening of Th2 allergic responses. In addition, HDM-stimulated splenocytes from sensitized IL-33 KO mice produced significantly less IL-4 (**Fig. 4E**), a critical Th2 cytokine in HDM allergy [10]. Lastly, we assessed the expression of OX40L on CD11b^+^ DCs in the lung (**Fig. 4F–G**). The proportion and numbers (not shown) of OX40L^+^ DCs were clearly elevated in WT mice exposed to Ad/GM-CSF+0.2 but not in IL-33 KO mice, which had comparable levels to the PBS+saline controls.

Similar results were observed in BALB/c ST2 KO mice (see **Fig. S2**); ST2 being the IL-33 receptor [11]. Collectively, these data show that GM-CSF-induced allergic sensitization and airway inflammation require IL-33 signaling.

### GM-CSF Induces IL-33 from Alveolar Type II Cells

**Figure 5:**
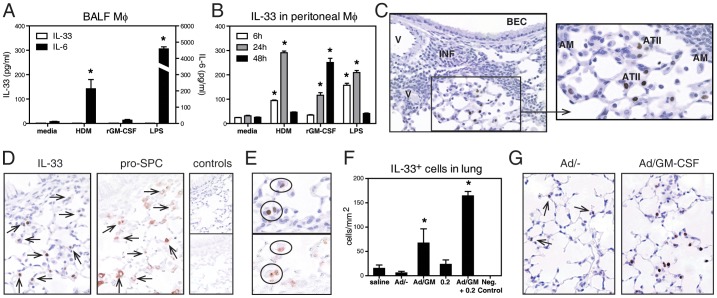
GM-CSF induces IL-33 on alveolar type II cells and peritoneal, but not alveolar, macrophages. (**A**) IL-33 and IL-6 by ELISA from freeze-thaw alveolar macrophages stimulated *in vitro* for 24 h. (**B**) IL-33 by ELISA from freeze-thaw peritoneal macrophages stimulated *in vitro* for 6, 24 or 48 h. Images and data in **C–G** are obtained at 2 wk. (**C**) Representative lung section upon exposure to Ad/GM-CSF+0.2, stained with anti-IL-33 Ab (nuclear dark brown stain), showing IL-33 immunostaining in alveolar walls but not in bronchial epithelial cells (BEC), inflammatory cells (INF) or vascular endothelial cells (V) (x200); magnified image (on the right) shows IL-33^-^ alveolar macrophages (AM) and IL-33^+^ alveolar type II cells (ATII). (**D**) Representative images (x400) from adjacent lung sections stained with either anti-IL-33 Ab (left image, dark brown in the nucleus) or anti-pro-SPC Ab (middle image, red in the cytoplasm) and negative controls for IL-33 (top right) and pro-SPC (bottom right). (**E**) Enlarged images. (**F**) IL-33^+^ cell counts in the lung of WT mice (or IL-33 KO as negative control). (**G**) Representative images showing distinct intensity of IL-33 stain in Ad/− *versus* Ad/GM-CSF exposed mice. N = 6 wells/condition in **A,B** or N = 3–7 mice/group in **E**. * p<0.05 versus media or saline.

Recent reports have suggested that alveolar macrophages are important sources of IL-33 in the lung upon helminth and viral infections [12], [13]. At variance with these observations, we found that IL-33 protein levels from alveolar macrophages stimulated with either HDM, recombinant GM-CSF (rGM-CSF) or LPS *in vitro* were below the limit of detection in both supernatants and cell lysates (**Fig. 5A**). These results were consistent at different time-points and at a range of doses (not shown). In contrast, alveolar macrophages stimulated under these conditions produced significantly increased IL-6 (**Fig. 5A**) and TNF-α (see **Fig. S3**). The ability of macrophages to upregulate IL-33 seems to be site-specific since peritoneal macrophages from naïve mice stimulated under identical conditions, including rGM-CSF, showed increased intracellular IL-33 (**Fig. 5B**). IL-33 was again undetected in supernatants (not shown).

IL-33 expression has been reported in lung epithelial cells and vascular endothelial cells of both mice and humans [11], [14]–[16]. Thus, we performed an immunohistochemistry analysis of lung sections with anti-IL-33 Ab. Images in **Fig. 5C** show positive and specific IL-33 staining of epithelial cells located in the alveoli but not conducting airways, inflammatory aggregates or vasculature. In agreement with the above *in vitro* data, alveolar macrophages did not stain for IL-33 (high-magnification image in **Fig. 5C**). IL-33^+^ cells were cuboidal and located at alveolar-septal junctions, suggesting these cells are airway epithelial type II cells, also known as type II pneumocytes or alveolar type II cells (ATII). As ATII cells specifically produce surfactant protein C (SPC) [17], we confirmed their identity by staining adjacent lung tissue sections with anti-pro-SPC Ab; indeed, all IL-33^+^ cells co-localized with pro-SPC^+^ cells (**Fig. 5D–E**). IL-33 was highly localized in the nucleus of ATII cells, and there was no evidence of cytoplasmic or extracellular localization. As expected, control slides demonstrated no IL-33 (**Fig. 5D**, top right image) or pro-SPC (**Fig. 5D**, bottom right image) staining. A quantitative analysis revealed a rare, scattered presence of weakly IL-33^+^ cells in lungs from mice exposed to saline, Ad/− or 0.2 µg HDM alone (**Fig. 5F–G**). In contrast, there were significantly more IL-33^+^ cells with more intense nuclear immunostaining in Ad/GM-CSF and Ad/GM-CSF+0.2 exposed mice (**Fig. 5F–G**). IL-33^+^ cells were primarily located in the upper and middle respiratory tract, and were rare in the lower lung (not shown), which parallels the regions of Ad infection and subsequent expression of GM-CSF. Thus, these data show that GM-CSF increases the number of IL-33-expressing ATII cells as well as the amount of nuclear IL-33 on a *per* cell basis.

### GM-CSF Modulates IL-33 Expression Independently of IL-1α

**Figure 6:**
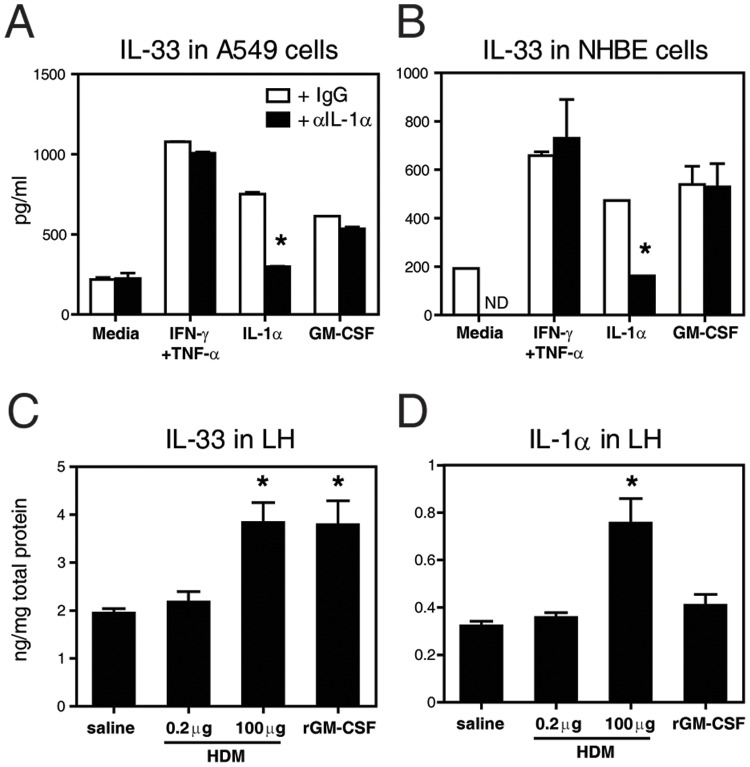
GM-CSF-driven IL-33 expression is IL-1α independent. (**A,B**) IL-33 by ELISA from freeze/thaw human A549 and NHBE cells stimulated *in vitro* for 24 h with or without neutralizing anti-IL-1α Ab. (**C**) IL-33 and (**D**) IL-1α by ELISA from lung homogenates of WT mice at 18 h after *in vivo* exposure to HDM or rGM-CSF. N = 5 mice/group. * p<0.05 versus saline. ND for not determined.

To determine whether GM-CSF could directly upregulate IL-33 in lung epithelial cells, *in vitro* assays were performed using the human ATII cell-derived cell line A549 and normal human bronchial/tracheal epithelial (NHBE) cells. **Fig. 6A–B** shows that intracellular IL-33 levels were increased upon stimulation with rGM-CSF. For comparison, cells were exposed to IFN-γ+TNF-α, as these cytokines have been shown to induce IL-33 expression in a variety of cell types [18], [19]. Willart *et al.* recently proposed that EC-derived IL-1α can act, in an autocrine manner, to induce GM-CSF and IL-33 production and release [20]. We found that whereas IL-1α was able to upregulate intracellular IL-33 in A549 and NHBE cell cultures, the levels of IL-33 induced by either rGM-CSF or IFN-γ+TNF-α stimulation were not affected by blockade of IL-1α using anti-IL-1α Ab. In addition, we found that *in vivo* administration of rGM-CSF readily elevated IL-33 in the lung while IL-1α levels remained comparable to those in saline-exposed mice (**Fig. 6C**). Lastly, we exposed separate groups of mice to either a very high dose (100 µg) or a sub-threshold dose (0.2 µg) of HDM *in vivo* and observed that only exposure to 100 µg HDM significantly increased IL-1α as well as IL-33 in the lung. Thus, these *in vivo* and *in vitro* results demonstrate that GM-CSF can increase intracellular IL-33 expression from lung epithelial cells, likely in an IL-1α independent manner.

## Discussion

The amount of inhaled HDM required to induce sensitization and, eventually, allergic disease in humans remains to be elucidated. As allergen exposure is universal but allergic asthma is not, it is likely that most individuals are exposed to sub-threshold concentrations. Allergen exposure rarely occurs in isolation, thus, understanding the impact of other environmental exposures on allergen responsiveness may shed light on the molecular requirements to develop allergic asthma. Here, we investigated whether and how environmental triggers that lead to GM-CSF production in the lung may facilitate the emergence of an allergic asthmatic phenotype upon exposure to sub-clinical concentrations of HDM.

We used GM-CSF as a surrogate environmental trigger because it is a powerful APC activator [5]–[7] produced in the lung in response to a variety of environmental stimuli, including bacterial and viral infections, allergens, fungi, cigarette smoke and pollutants [21]–[26]. Moreover, it has been shown that several polymorphisms in the GM-CSF gene are risk factors for the development of atopy and atopic diseases [27], [28]. Our data show that enhanced GM-CSF expression in the airways alters the local environment to promote cardinal immune, inflammatory, structural and functional features of the allergic asthmatic phenotype upon exposure to sub-threshold levels of allergen. We used a replication-defective adenoviral GM-CSF gene transfer approach as a vector system, instead of recombinant cytokines or transgenic mice, to induce extended, but transient, expression of GM-CSF. The dose of Ad vector used results in levels of GM-CSF in BALF (∼90 pg/ml) that can be considered physiologically relevant [25], [29], does not induce tissue damage and causes only very mild lung inflammation consisting of mononuclear cells but not eosinophils. Importantly, 3×10^7^ pfu of the control viral vector did not cause any detectable immune-inflammatory change in the lung.

We have previously shown that continuous exposure to amounts of allergen below 2 µg does not cause detectable inflammatory changes in the lung, even after five months [3]. Here, we show that a GM-CSF enriched lung environment increases the sensitivity and reactivity of the host to HDM. Strikingly, about ten times less allergen was needed to trigger eosinophilic inflammation and goblet cell hyperplasia/hypertrophy with mucus production. These changes were associated with activation of the adaptive immune system, as mice exposed to the sub-threshold dose of 0.2 µg HDM in the context of GM-CSF had significantly more activated Th2 and B cells that produced high levels of IL-5 and IL-13, total IgE, HDM-specific IgG1 and, later, HDM-specific IgE, in accordance with the progression of isotype class switching. Our data show that GM-CSF alone and, to a greater extent, in combination with 0.2 µg HDM, expanded CD11c^+^MHCII^hi^ lung DCs. In addition, most CD11c^+^MHCII^hi^ cells were also CD11b^+^ and exhibited upregulated expression of the co-stimulatory molecule CD86. These cells have been shown to be necessary and sufficient for the initiation of Th2 responses to HDM [30]; they are rapidly recruited to the lungs during HDM exposure and can migrate to draining lymph nodes [21].

The early immune-inflammatory responses described above were antigen-specific and had long-term consequences, as only mice initially sensitized with Ad/GM-CSF+0.2 exhibited, one month later, eosinophilic inflammation in response to a brief re-exposure with 0.2 µg HDM, and even 0.02 µg HDM. At this time point, high levels of HDM-specific IgG1 and IgE were detected. Lung dysfunction (AHR) was also apparent after extended allergen exposure, with increased airway and tissue resistance to methacholine challenge indicating higher broncho-constriction and heterogeneity of small airway narrowing. Collectively, the data show that incipient expression of GM-CSF in the airway expanded and activated CD11b^+^ DCs in the lung, thus favouring the induction of an adaptive immune response to otherwise innocuous amounts of HDM.

IL-33 is a novel member of the IL-1 family of cytokines [11]. Higher levels of IL-33 have been detected in the lung of asthmatics [14], [18], and genetic analyses have linked polymorphisms in the *il-33* and *st2* genes with allergic disease and asthma [31]. Overexpression of IL-33 in transgenic mice or administration of recombinant IL-33 leads to Th2 responses [9], [32], [33]. However, discrepant results have been reported in regards to the relevance of IL-33 signaling in promoting allergic responses in ovalbumin-based experimental models [34]–[37]. We have recently reported that IL-33 plays, along with the co-stimulatory molecule OX40L, a central role in allergic sensitization and inflammation in a HDM-driven system [9]. However, the molecular signals that trigger IL-33 production remain unclear. Here, we show for the first time that GM-CSF increases the levels of IL-33 protein in the lung, and that GM-CSF-driven allergic responses are mediated, at least in part, by IL-33 signalling. Indeed, eosinophilic inflammation, IL-4 production and OX40L upregulation on DCs were markedly decreased in mice deficient in IL-33 signalling. IL-33 can be produced by a number of cell types, such as bronchial epithelial and vascular endothelial cells in both humans and mice [11], [14], [16]. Also, alveolar macrophages can express IL-33 in response to influenza A infection [13] as well as to some [12] but not other [38] helminth infections. Recent studies have found that ATII cells are the major source of IL-33 at baseline and upon lung inflammation following exposure to OVA, ragweed, papain as well as upon certain fungal and helminth infections [38]–[41]. We show that GM-CSF expression in the lung led to increased numbers of IL-33^+^ cells. These cells had morphological features of ATII cells and were also pro-SPC^+^, a specific ATII cell marker [17]. We did not find IL-33^+^ alveolar macrophages, nor did they produce IL-33 upon stimulation with rGM-CSF, HDM or LPS *in vitro*. Interestingly, we found that peritoneal macrophages increased intracellular IL-33 upon HDM, rGM-CSF or, as previously published [42], LPS stimulation. These data suggest that GM-CSF can directly activate ATII cells to produce IL-33. From a broader perspective, it seems increasingly clear that the cellular source of IL-33 is dependent on both the site and the context. Further studies should elucidate whether ATII cells are the dominant source of IL-33, as well as IL-33′s importance, at later stages of disease progression.

Many questions regarding the mechanisms of processing and secretion of IL-33 remain unsolved. IL-33 is usually found in the nucleus, where it is thought to act as a nuclear factor, specifically as a histone-binding factor that regulates gene expression [16], [43]. In agreement with previous reports [38], [39], we detected only nuclear IL-33. However, it is clear that IL-33 can also function extracellularly as a cytokine that activates target cells expressing the ST2 receptor. Indeed, our results in IL-33 KO and ST2 KO mice indicate that IL-33/ST2 interactions drive allergic sensitization to HDM and, thus, IL-33 is important as a released cytokine. It is likely that rapid degradation after release, by a mechanism yet to be clarified, accounts for the difficulties in measuring IL-33 in fluids.

It has been recently proposed that HDM stimulates epithelial cells to release IL-1α which, then, acts in an autocrine manner to release GM-CSF and IL-33 that mediate DC recruitment and activation [20]. It should be pointed out that these findings were made in a system solely driven by HDM, involving, in some instances, exposure to exceptionally high doses of HDM (100 µg intratracheally). We found that administration of rGM-CSF *in vivo* enhanced IL-33 but not IL-1α levels in the lung. In addition, *in vitro* assays using human A549 and NHBE cells demonstrated that GM-CSF-driven induction of intracellular IL-33 expression is IL-1α independent. Thus, in the likely common real-life scenario of exposure to sub-threshold amounts of allergen, our data propose a novel molecular pathway of Th2 immunity that is independent of IL-1α and where GM-CSF is upstream of IL-33.

**Figure 7:**
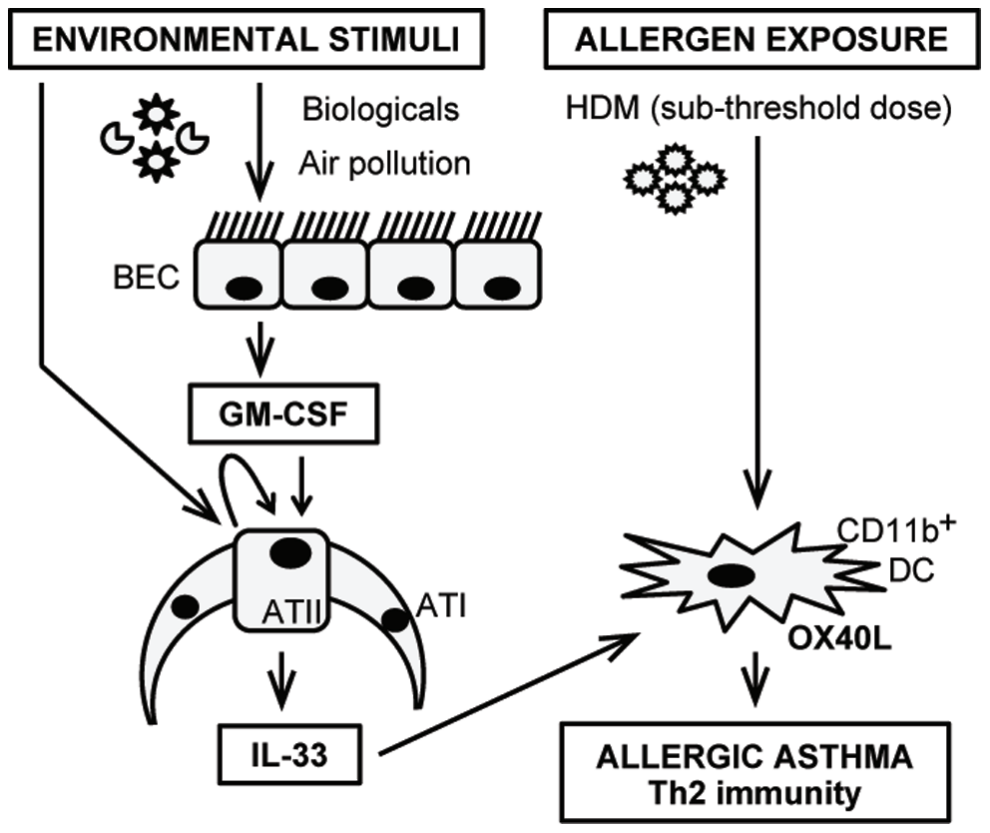
Schematic of the proposed model. Exposure to a variety of environmental stimuli can result in GM-CSF production from bronchial epithelial cells (BEC) and alveolar epithelial type II cells (ATII). GM-CSF induces IL-33 production from ATII cells in an IL-1α-independent manner. Then, IL-33 acts locally on CD11b^+^ dendritic cells (DC), which reside in close proximity to epithelial cells, to upregulate OX40L and enhance antigen presentation. Such activated status of the lung immunological environment facilitates allergic sensitization to even sub-clinical amounts of inhaled allergens and, ultimately, results in the development of allergic asthma.

In conclusion (**Fig. 7**), we have demonstrated that an initial and transient immune perturbation in the lung, namely GM-CSF expression, lowers the threshold of immune responsiveness to HDM and facilitates robust allergic inflammatory responses. We also show that GM-CSF induces intracellular IL-33 expression from ATII cells, and that immune-inflammatory responses to sub-threshold amounts of HDM in the context of GM-CSF expression, including OX40L upregulation, are substantially IL-33 dependent. These data suggest that in a model of allergic asthma that is not driven by the allergen (HDM) itself, a GM-CSF/IL-33/OX40L pathway facilitates the development of an allergic asthmatic phenotype. This pathway may be particularly relevant where allergic asthma is precipitated by environmental exposures, such as certain infections and pollution.

## Materials and Methods

### Animals

Female BALB/c mice (6–8 weeks old) were purchased from Charles River Laboratories and Taconic. IL-33 KO and ST2 KO [36] mice were kindly provided by MedImmune (Gaithersburg, MD) from Taconic (Derwood, MD) and Charles River Laboratories (Frederick, MD), respectively. Mice were housed in a specific pathogen-free environment. All experiments were approved by the Animal Research Ethics Board of McMaster University.

### Adenovirus Administration

A replication-deficient human type 5 adenoviral construct carrying mouse GM-CSF cDNA in the E1 region of the viral genome (Ad/GM-CSF) was delivered intranasally. Ad/GM-CSF or an E1-deleted control virus (Add170-3 or Ad/−) was administered at a dose of 3×10^7^ pfu in 30 µl PBS, as previously described [6], [7].

### House Dust Mite Administration

Mice received 5-fold increasing doses (0.2, 1, 5 or 25 µg) of HDM (*Dermatophagoides pteronyssinus*, Greer Laboratories) in 10 µl saline once daily for 7 consecutive days or 5 days/week followed by 2 days of rest for a total of 2 or 4 weeks, as specified. For the *in vivo* recall protocol, mice were exposed to 0.2 µg HDM for 10 consecutive days, then rested for 4 weeks, and re-exposed to saline, 0.02 or 0.2 µg HDM for 3 consecutive days. To evaluate very early events, mice received only one administration of 0.2 or 100 µg HDM, or 4 µg recombinant murine GM-CSF.

### Sample Collection

Mice were killed 24 h after the last exposure to HDM, unless otherwise specified, and blood, spleens and thoracic lymph nodes were collected. The **lungs** were also dissected and two lavages (0.25+0.2 ml) were done with PBS supplemented with complete protease inhibitor (Roche). Cytospins were prepared and stained with Protocol Hema 3 set (Fisher Scientific) and ∼500 cells were counted and identified as mononuclear cells, neutrophils and eosinophils. Following BALF, the left lobe of the lung was slowly inflated and fixed in 10% formalin for histological analysis. The right lobes were homogenized as previously described [9]. Alternatively, lungs were perfused with PBS and left and/or right lobes were kept in ice-cold HBSS until processing for flow cytometric analysis.

### Cell Cultures

**Splenocytes** were isolated and resuspended to 8×10^6^ viable cells/ml in complete RPMI, as previously described [9]. Cells were cultured in triplicate in medium alone or with 15.625 µg/ml HDM in flat-bottom, 96-well plates for 5 days. **Alveolar and peritoneal macrophages** were recovered from the lungs and peritoneal cavity of naïve mice with ice cold PBS supplemented with 0.5 mM EDTA and, then, washed with complete RPMI. 0.5−1×10^5^ macrophages/well were plated and allowed to adhere at 37°C for 1 h. Non-adherent cells were removed by gently washing three times with warm PBS. Macrophages were stimulated with or without increasing doses of HDM (up to 100 µg/ml), recombinant murine GM-CSF (up to 1 µg/ml) or LPS (up to 100 ng/ml; Sigma-Aldrich) for 2, 6, 24, 48 or 72 h. For **A549 cells** (ATCC) and **normal human bronchial/tracheal epithelial cells** (NHBE, Lonza), 1−2×10^5^ cells were allowed to adhere overnight in either DMEM supplemented with 10% FBS and penicillin/streptomycin, or Bronchial Epithelial Growth Medium BulletKit (Lonza), respectively. The following day, cells were primed with recombinant human IFN-γ (50 ng/ml), TNF-α (10 ng/ml), IL-1α (50 ng/ml) or GM-CSF (50 ng/ml) (all from R&D Systems) for 24 h with or without neutralizing anti-IL-1α Ab (10 µg/ml; R&D). Supernatants from both macrophages and epithelial cells were collected and fresh media was added to cells prior to 3–5 cycles of freeze/thawing.

### Cell Isolation and Flow Cytometric Analysis

**Lung cells** were isolated by collagenase digestion (Collagenase type I, Gibco-Life Technologies) and passed through 40 µm cell strainers, as previously described [9]. **Lymph nodes** were triturated between frosted slides in HBSS. All cells were washed in FACS buffer (0.5–1% BSA and 10 mM EDTA-supplemented PBS) and filtered again before staining. For each antibody combination, 2–4×10^6^ cells were first incubated with anti-CD16/CD32 (eBioscience) and then stained for 30 min at 4°C. Antibodies were obtained from BD Biosciences, eBioscience, BioLegend or MD Biosciences and titrated before use. Propidium iodide (Sigma-Aldrich) and forward scatter/side scatter were used to exclude dead cells and doublets. Data were acquired on a BD LSR II and analyzed with FlowJo software (TreeStar). Fluorescence minus one controls were used for gating.

### H&E and PAS Histology

Paraffin sections of lungs were stained with H&E or PAS, as previously described [44]. For PAS stain quantification, multiple images (up to 15) of main airways were captured with OpenLab software (version 5.5.0; Improvision) via a MicroPublisher camera (5.0 RTV; QImaging) and Leica microscope. Morphometric analysis was performed using a custom computerized analysis system (Northern Eclipse software version 5; Empix Imaging) that calculates the percentage of tissue area that was positively stained within a 30 µm wide band from the basement membrane extending into the airway lumen.

### Immunohistochemistry

Paraffin sections of lungs were deparaffinized in xylene, and hydrated through graded alcohols. To stain for IL-33, heat-induced epitope retrieval was performed using 10 mM citrate buffer and, then, sections were placed in 0.05 M Tris-HCl-Tween buffer. We blocked endogenous peroxidase with 3% H_2_O_2_ in distilled water, and proteins with 1% BSA. Sections were then incubated with anti-IL-33 Ab (1∶50; monoclonal biotinylated Ab, Nessy-1, Enzo Life Sciences) in Tris+Tween+saponin buffer for 1 h. Finally, we used streptavidin/peroxidase conjugate (Dako) and prepared DAB chromogenic substrate. To stain for pro-SPC, endogenous peroxidase was blocked with 3% H_2_O_2_ in methanol, with HCl added last. Proteinase K (Dako) was used to unmask antigens/epitopes, and proteins were blocked with 5% normal goat serum. Sections were incubated with anti-pro-SPC Ab (1∶2000; polyclonal Ab, Millipore) in Ultra Clean Diluent (Fisher Scientific) for 1 h. Finally, we used EnVision detection system with DAB+ (Dako). Mayer’s hematoxylin was used to counterstain. Negative controls were generated by omitting the primary Ab. For enumeration of IL-33^+^ cells, counts were made from five fields of view *per* mouse (2 from the upper lobe, 2 from the middle and 1 from the lower respiratory tract) at 400× and normalized per unit area (mm^2^).

### Cytokine and Ig Analysis

Murine GM-CSF, IL-33, IL-6, TNF-α and IL-1α as well as human IL-33 were measured by ELISA (DuoSet kits, R&D Systems), and murine IL-4, IL-5, IL-10, IL-13, IFN-γ and TNF-α by Luminex (multiplex kits, Millipore) according to manufacturer’s protocols. Total protein content was quantified by using the Bradford assay (Bio-Rad Laboratories). Total or HDM-specific Ig levels were measured by using ELISA techniques, as previously described [9], [45]. HDM-specific Ig levels are depicted as OD – background, where background = average OD of at least 20 blank wells+two standard deviations.

### Airway Responsiveness

Mice were anesthetized, paralyzed, tracheostomized and mechanically ventilated with a small animal computer-controlled piston ventilator (flexiVent, SCIREQ Inc.). The response to nebulized saline and increasing doses (3.125, 12.5 and 50 mg/ml) of methacholine (MCh, Sigma-Aldrich) was measured as previously described [3]. Model fits that resulted in a coefficient of determination less than 0.8 were excluded.

### Data Analysis

Data are expressed as mean ± standard error of the mean and were analyzed and graphed with Prism software version 5 (GraphPad). Shown are either pooled data from 2–3 experiments yielding similar results or 1 representative experiment of 2–4. Statistical analysis was calculated using Student’s *t* test (unpaired, two-tailed) and one- or two-way analysis of variance (with Bonferroni *post hoc* test). Differences were considered statistically significant when p<0.05. For some outcomes, the experimental data was best fitted with a sigmoidal dose-response function using Prism.

## Supporting Information

Figure S1 **GM-CSF overexpression changes the nature and activation status of APCs in the lung.** (A) Gating strategy for DCs. (B) Proportion of DCs (CD45^+^CD11c^+^MHCII^hi^B220^-^Gr1^-^). (C) Numbers of activated CD11b^+^ DCs (CD45^+^CD11c^+^CD11b^+^MHCII^hi^CD86^+^). (D) Representative flow plots of CD11b^+^ DCs showing expression of MHCII and CD86. All samples were collected at day 7, i.e. at a time when GM-CSF levels are elevated. N = 3–5 mice/group. * p<0.05 in Ad/GM-CSF+saline/HDM versus respective PBS control.
(EPS)

Figure S2 **Attenuated allergic responses in the absence of IL-33 receptor.** (A–C) BALB/c WT and ST2 KO mice were exposed to the 2 wk protocol (as shown in Figure 1B). (A) Total cells and (B) eosinophil numbers and percentages in BALF. (C) IL-5 production by splenocytes stimulated *ex vivo* with HDM. N = 2–9 mice/group and * p<0.05 in ST2 KO versus respective WT.
(EPS)

Figure S3 **Alveolar macrophages do not produce TNF-α upon rGM-CSF stimulation.** TNF-α by ELISA from freeze-thaw alveolar macrophages stimulated *ex vivo* for 24h. N = 6 wells/condition and * p<0.05 versus media.
(EPS)
